# Effect of happiness and functional disability on older people’s survival

**DOI:** 10.1590/0102-311XEN054624

**Published:** 2025-03-24

**Authors:** Donatila Barbieri de Oliveira Souza, Luciana Correia Alves, Marilisa Berti de Azevedo Barros, Bruna Kelly Fehlberg, Margareth Guimarães Lima

**Affiliations:** 1 Faculdade de Ciências Médicas, Universidade Estadual de Campinas, Campinas, Brasil.; 2 Instituto de Filosofia e Ciências Humanas, Universidade Estadual de Campinas, Campinas, Brasil.

**Keywords:** Happiness, Survival, Aged, Functional Status, Felicidade, Sobrevida, Idoso, Capacidade Funcional, Felicidad, Sobrevida, Anciano, Estado Funcional

## Abstract

The aim was to investigate survival and risk of death within a ten-year period according to physical functioning and frequency of the feeling of happiness in older people, conducting an analysis of the possible mediating effect of happiness on the association between physical functioning and mortality. A retrospective longitudinal study was conducted with 1,519 older people (≥ 60 years) interviewed for the 2008/2009 *Health Survey in Campinas*. A linkage was made between the databank of the survey and the Campinas Mortality Information System, with active search for confirmation of deaths and non-deaths from 2008 to 2018. Variables of interest were physical functioning (absence/presence of limitations) and frequency of feeling happiness. Kaplan-Meier survival curves were plotted and Cox regression analysis was performed to estimate hazard ratios (HR). A mediation analysis was also conducted using the Karlson-Holm-Breen (KHB) method. In the adjusted analysis, severe functional limitation (HR = 2.8; 95%CI: 2.0-3.8) and low frequency of happiness (HR = 1.6; 95%CI: 1.3-2.0) increased the risk of death in the period. Low frequency of happiness mediated the association between functioning and mortality by 14%. The results underscore the importance of strategies to maintain physical functioning during aging. Moreover, a greater frequency of the feeling of happiness increased the survival of the population. The findings also show that happiness plays an important mediating role in the association between functioning and mortality in older people.

## Abstract (pt)

O objetivo deste estudo foi investigar a sobrevivência e o risco de morte em um período de 10 anos de acordo com a funcionalidade física e a frequência do sentimento de felicidade em pessoas idosas, além de realizar uma análise do possível efeito mediador da felicidade na associação entre funcionalidade física e mortalidade. Foi realizado um estudo longitudinal retrospectivo com 1.519 pessoas idosas (≥ 60 anos) entrevistadas para o *Inquérito de Saúde de Campinas* de 2008-2009. Foi realizado um *linkage* entre o banco de dados da pesquisa e o Sistema de Informação sobre Mortalidade de Campinas, com busca ativa para a confirmação de óbitos e não óbitos de 2008 a 2018. As variáveis de interesse foram a funcionalidade física (ausência/presença de limitações) e a frequência do sentimento de felicidade. Foram traçadas curvas de sobrevivência de Kaplan-Meier e realizada a análise de regressão de Cox para o cálculo das razões de risco (RR). Também foi realizada uma análise de mediação utilizando o método Karlson-Holm-Breen (KHB). Na análise ajustada, a limitação funcional grave (RR = 2,8; IC95%: 2,0-3,8) e baixa frequência de felicidade (RR = 1,6; IC95%: 1,3-2,0) aumentaram o risco de óbito no período. A baixa frequência de felicidade mediou a associação entre funcionalidade física e mortalidade em 14%. Os resultados destacam a importância de estratégias voltadas para a manutenção da funcionalidade física durante o processo de envelhecimento. Além disso, uma maior frequência do sentimento de felicidade aumentou a sobrevivência da população. Os resultados também mostram que a felicidade desempenha um papel importante de mediação na associação entre capacidade funcional e mortalidade em pessoas idosas.

## Abstract (es)

El objetivo de este estudio fue investigar la supervivencia y el riesgo de muerte en un período de 10 años según la funcionalidad física y la frecuencia del sentimiento de felicidad en personas mayores, además de realizar un análisis del posible efecto mediador de la felicidad sobre la asociación entre la funcionalidad física y la mortalidad. Se realizó un estudio longitudinal retrospectivo con 1.519 personas mayores (≥ 60 años) participantes en la *Encuesta de Salud de Campinas* de 2008-2009. Se realizó una vinculación entre la base de datos de investigación y el Sistema de Información de Mortalidad de Campinas, con una búsqueda activa de la confirmación de defunciones y no defunciones de 2008 a 2018. Las variables de interés fueron la funcionalidad física (ausencia/presencia de limitaciones) y la frecuencia del sentimiento de felicidad. Se trazaron curvas de supervivencia de Kaplan-Meier, y se realizó un análisis de regresión de Cox para calcular las razones de riesgo (RR). También se realizó un análisis de mediación utilizando el método Karlson-Holm-Breen (KHB). En el análisis ajustado, la limitación funcional severa (RR = 2,8; IC95%: 2,0-3,8) y la baja frecuencia de felicidad (RR = 1,6; IC95%: 1,3-2,0) aumentaron el riesgo de muerte en el período. La baja frecuencia de felicidad favoreció la asociación entre la funcionalidad física y la mortalidad en un 14%. Los resultados destacan la importancia de las estrategias dirigidas a mantener la funcionalidad física durante el proceso de envejecimiento. Además, una mayor frecuencia del sentimiento de felicidad aumentó la supervivencia de la población. Los resultados también muestran que la felicidad juega un importante papel mediador en la asociación entre la capacidad funcional y la mortalidad en las personas mayores.

## Introduction

Aging is associated with an increased prevalence of chronic noncommunicable diseases ^1^ ^,^ ^2^ and reduced physical functioning ^3^ ^,^ ^4^, which lead to a decreased well-being ^5^ and quality of life ^6^, as well as a greater risk of premature death ^7^ ^,^ ^8^. 

Functional disability is an acquired difficulty in performing basic activities of daily living (ADLs) or more complex tasks that are fundamental to an independent life ^9^. The prevalence and incidence of functional disabilities in Brazil has been the object of research over time ^10^ ^,^ ^11^ ^,^ ^12^ ^,^ ^13^.

Data from the 2013 and 2019 *Brazilian National Health Survey* (PNS, acronym in Portuguese) provide comprehensive and representative information on the prevalence of functional disabilities in Brazil. In the 2013 PNS, the total prevalence of disabilities regarding ADLs among people aged 60 years or older was 15.8% ^14^ ^,^ ^15^. The 2019 PNS reported a similar prevalence, highlighting the persistence of functional disabilities in the aging population ^16^ ^,^ ^17^. National and international studies report rates ranging from 12.0% to 36.1% ^18^ ^,^ ^19^ ^,^ ^20^ ^,^ ^21^. Recent Brazilian studies have expanded the understanding of functional disabilities among older adults ^22^. Alexandre et al. ^22^ showed that the incidence of disabilities was 44.7/1,000 persons/year for women and 25.2/1,000 persons/year for men.

The relationship between functional disability and mortality in older people has been addressed in national ^23^ ^,^ ^24^ ^,^ ^25^ and international ^26^ ^,^ ^27^ ^,^ ^28^ ^,^ ^29^ studies. Morbidities, in addition to being the main cause of death in this age group ^30^, considerably influence physical functioning ^31^. However, disabilities can increase the risk of premature death due to limited mobility, causing changes in the metabolism and functioning of the cardiovascular, respiratory, musculoskeletal and gastrointestinal systems ^32^ ^,^ ^33^. Moreover, disabilities can reduce well-being by causing dependence, restricting autonomy and individual freedom, and affecting social relations ^34^. These factors have a substantial effect on satisfaction with life and can diminish well-being and happiness ^5^ ^,^ ^35^. However, we found no studies exploring the extent to which the feeling of happiness may mediate the association between functional disability and mortality.

Happiness is the degree to which an individual favorably assesses the overall quality of their life ^36^. A low frequency of happiness is also correlated with an increased risk of premature death ^37^. Longitudinal studies have stressed the importance of happiness and optimism in preventing morbidities ^38^, adopting healthier behaviors ^39^ and increasing longevity ^40^ ^,^ ^41^. Thus, there is a need to clarify associations between the feeling of happiness and health conditions. A deeper understanding of underlying mechanisms will enable knowledge that can guide clinical strategies and policies that seek to ensure wellbeing and extend longevity in the older population.

This study aimed to investigate survival and risk of death within a 10-year period and associate these aspects with functional disability and happiness in older people, analyzing the possible mediating effect of happiness in the association between physical functioning and mortality. Previous studies indicate that the impact of functionality and happiness on mortality can vary significantly between men and women, as well as across different age groups ^42^ ^,^ ^43^. Stratified analyses by sex and age were conducted to identify specific patterns within these subpopulations (Campinas, São Paulo State, Brazil, 2008-2018).

## Material and methods

A retrospective, longitudinal study (ISA-Camp Cohort) was carried out using data from the *Health Survey in Campinas* (ISA-Camp), conducted from 2008 to 2009, in which 1,519 older people were interviewed. The 2008/2009 ISA-Camp had a complex sampling design with probabilistic cluster sampling in two stages: census sectors and households ^44^. A total of 50 census sectors were randomly selected with probability proportional to the number of households, followed by the systematic selection of households in each selected census sector. These sectors were ordered by the percentage of heads of households with complete tertiary education.

Databanks of the Brazilian Mortality Information System (SIM, acronym in Portuguese) of the Campinas Municipal Health Department were searched to obtain information on older people who had answered questionnaire of the 2008/2009 ISA-Camp and died between 2008 and 2018. Deterministic, probabilistic linkage was performed in the databanks with the Stata 15.0 statistical program (https://www.stata.com) considering name, sex and date of birth. Paired records denoted deaths and unpaired records denoted non-deaths. To confirm non-deaths, an active search (telephone contact) was conducted. When such contact was not possible, visits to the households were made to confirm the status of the individual (death/non-death).

The interviewers received training, which involved meetings and orientation on how to communicate with residents via telephone and in-person, as well as with family members and acquaintances (when the individual was not located), to minimize losses. Individuals not found after three attempts at telephone contact and three unsuccessful visits were considered losses and excluded from the analyses. A total of 219 losses occurred − 202 individuals were not found; information was incomplete for six deaths (missing year of death) and 11 individuals did not answer the *Short Form* (36) *Health Survey* (SF-36) questionnaire. The final sample consisted of 1,300 individuals, of which 855 remained alive and 445 died.

### Variables

The dependent variable was time until death, which was determined by subtracting the date of death from the date of the interview. Information on non-death up to December 31st, 2018 was censored. 

Primary independent variables were functional disability (no disability, mild/moderate limitation or severe limitation) and subjective well-being (frequency of feeling of happiness). Functional disability and happiness are the main exposures of interest. Additionally, sex, age (60-74 years and 75 years or older), schooling (0-3, 4-7, and 8 or more years of study), and the number of chronic diseases (none, one, and two or more) were included in the model for adjustment purposes.

Indicators on functional disability used in this study were walking more than one kilometer (F03g), walking several blocks (several hundreds of meters) (F03h), walking one block (100 meters) (F03i) and bathing or dressing (F03j). These four items are part of the SF-36 questionnaire ^45^ and these indicators were also used on previous Brazilian studies with similar items to classify different degrees of functional disability ^46^ ^,^ ^47^. Individuals who answered “yes” to items F03g and/or F03h but “no” to items F03i and/or F03j were considered to have mild/moderate limitations. Those who answered “yes” to items F03i and F03j were considered to have severe limitations. For analysis purposes, these categories were combined, and participants were classified as follows: (0) without disability, (1) mild/moderate or (2) severe functional disability. Dichotomous variables (0) without disability and (1) with disability (encompassing mild/moderate and severe disability) were used for mediation analysis.

The indicator of frequency of the feeling of happiness was the answer to the following question: “How often have you felt happy in the last four weeks?” Response categories were dichotomized as “always” or “most of the time” (scored 0, greater frequency of happiness) and “a small part of the time” or “never” (scored 1, lower frequency of happiness). This indicator has been used ^48^ ^,^ ^49^ ^,^ ^50^ ^,^ ^51^ to assess subjective well-being and is considered a valid and direct measure of happiness ^5^ ^,^ ^41^. For analysis purposes, participants were categorized as having a higher or lower frequency of happiness based on their responses. All independent variables were collected at baseline. 

### Data analysis

Percentages of deaths in the period were estimated according to physical functioning and feeling of happiness in the overall sample, as well as stratified by sex and age group. Survival analyses were performed with the Kaplan-Meier estimator according to physical functioning and well-being. The log-rank test was used to compare the curves, considering a 5% significance level. Cox semiparametric regression models were run for the estimates of associations between each independent variable and dependent variables, estimating hazard ratios (HR).

Odds ratios (OR) were also estimated using multiple binary logistic regression to determine associations between mortality and both functional disability and the feeling of happiness. Mediation analysis was then conducted using happiness as a possible mediator of the association between functional disability and mortality. The Karlson-Holm-Breen (KHB) method ^52^ with a 5% significance level was used to estimate total and direct effects of functional disability on mortality, as well as the mediation percentage. The KHB method decomposes the OR and estimates the percentage of the mediator in the association ^52^. We previously tested possible interaction effects of mediator versus exposure in the association and did not find significant results at the 5% level.

To control for confounding, the following variables were incorporated into the multiple regression and KHB models: sex, age, schooling and chronic diseases. The choice of these adjustment variables was made based on the analysis of directed acyclic graphs ^53^. 

The Stata 15.0 software program was used. Cox regression, logistic regression, and KHB analyses were conducted using the survey module, which incorporates the complex sampling plan of the 2008/2009 ISA-Camp. The analysis of residuals was performed to determine the goodness-of-fit of the Cox model. Schoenfeld’s test and global proportionality analyses were performed. 

### Ethical aspects

The 2008/2009 ISA-Camp and ISA-Camp Cohort study received approval from the Human Research Ethics Committee of the School of Medical Sciences of the University of Campinas (approval n. 079/2007 and 1.650.093/2018, respectively). This study received approval from the local research ethics committee (n. 6.276.539/2023).

## Results

Median age was 69 years in the overall sample (n = 1,300). A total of 61.7% of the 855 living individuals were women. A total of 52% of participants were aged 60-69 years; 79.9% self-declared as white; 82.3% had a monthly income of less than three minimum wages; and 70.8% had less than eight years of schooling (data not presented).

Table 1 shows the mortality rate was 27.3% (95%CI: 24.1-30.8) among individuals without functional disability, 39.8 (95%CI: 33.7-46.2) among those who reported mild to moderate limitation and 65.4% (95%CI: 55.3-74.3) among those who reported severe functional disability. Regarding the frequency of the feeling of happiness, the mortality rate was 30.6% (95%CI: 26.9-34.5) among those who reported feeling happy most of the time/always and 45.7% (95%CI: 39.9-51.5) among those who reported feeling happy only a small part of the time/never. These results show a dose-response gradient for both functional disability and the feeling of happiness.

**Table 1:** Percentage of deaths according to physical functioning and feeling of happiness. *Health Survey in Campinas Cohort* (ISA-Camp Cohort), 2008-2018.

Variables	n	Deaths (%)	95%CI
Physical functioning			
No limitation	847	27.34	24.12-30.83
Mild to moderate limitation	319	39.84	33.76-46.26
Severe limitation	134	65.44	55.35-74.31
Frequency of feeling of happiness			
Most of the time or always	984	30.59	26.93-34.51
Small part of the time or never	316	45.66	39.92-51.53
Sex			
Male	535	38.64	33.88-43.61
Female	765	30.79	27.74-34.01
Age (years)			
60-69	676	20.16	16.82-23.98
70-74	250	36.04	29.72-42.89
75 or older	374	58.15	52.84-63.27
Education level (years)			
0-3	474	41.02	36.33-45.88
4-7	445	34.36	29.68-39.36
8 or more	380	26.05	21.14-31.64
Number of chronic diseases			
None	228	29.07	22.63-36.47
One	286	28.61	23.33-34.55
Two or more	766	37.61	34.25-41.09

95%CI: 95% confidence interval.

Source: Brazilian Mortality Information System (SIM) of the Campinas Municipal Health Department.

In the analyses stratified by sex and age group, we observed that the mortality rate was higher among men with no limitations (31.7%; 95%CI: 26.8-37.1) compared to women with no limitations (23.2%; 95%CI: 19.7-27.2). On the other hand, no difference between sexes was found among individuals with severe limitation when considering confidence intervals. Notably, this finding suggests that the distinct experience of mortality between sexes disappears when considering individuals with severe limitations. The same pattern was not observed in the analysis of the feeling of happiness. In the younger age group (60-74 years), severe limitations substantially increased the mortality rate among men and women compared to those without limitations. In the older age group (75 years or older), severe limitations increased the mortality rate only among women. Considering the category of lower frequency of happiness in both age groups, the mortality rate was higher only among women (Table 2). 

**Table 2:** Percentage of deaths according to physical functioning and feeling of happiness stratified by sex and age. *Health Survey in Campinas Cohort* (ISA-Camp Cohort), 2008-2018.

Variables	Male		Female	
	Deaths (%)	95%CI	Deaths (%)	95%CI
Total				
Physical functioning				
No limitation	31.73	26.77-37.15	23.23	19.68-27.21
Mild to moderate limitation	51.86	43.01-60.60	32.98	25.91-40.92
Severe limitation	74.68	60.09-85.24	61.74	50.08-72.2
Frequency of feeling of happiness				
Most of the time or always	36.61	31.27-42.30	25.59	21.82-29.76
Small part of the time or never	46.43	36.59-56.56	45.20	38.34-52.25
60-74 years				
Physical functioning				
No limitation	23.52	18.86-28.92	19.50	15.97-23.60
Mild to moderate limitation	35.71	25.00-48.08	21.94	15.67-29.83
Severe limitation	62.93	42.03-79.91	41.88	26.12-59.50
Frequency of feeling of happiness				
Most of the time or always	25.66	20.75-31.27	18.35	14.37-23.13
Small part of the time or never	34.57	25.44-45.01	33.02	24.40-42.96
75 years or older				
Physical functioning				
No limitation	63.87	52.96-73.52	35.89	27.92-44.72
Mild to moderate limitation	76.89	65.11-85.57	49.90	37.13-62.68
Severe limitation	87.66	62.08-96.86	78.95	64.64-88.51
Frequency of feeling of happiness				
Most of the time or always	69.78	60.27-77.86	42.66	35.15-50.52
Small part of the time or never	73.67	58.45-84.78	67.63	56.03-77.41

95%CI: 95% confidence interval.

Source: Brazilian Mortality Information System (SIM) of the Campinas Municipal Health Department.

**Figure 1:**
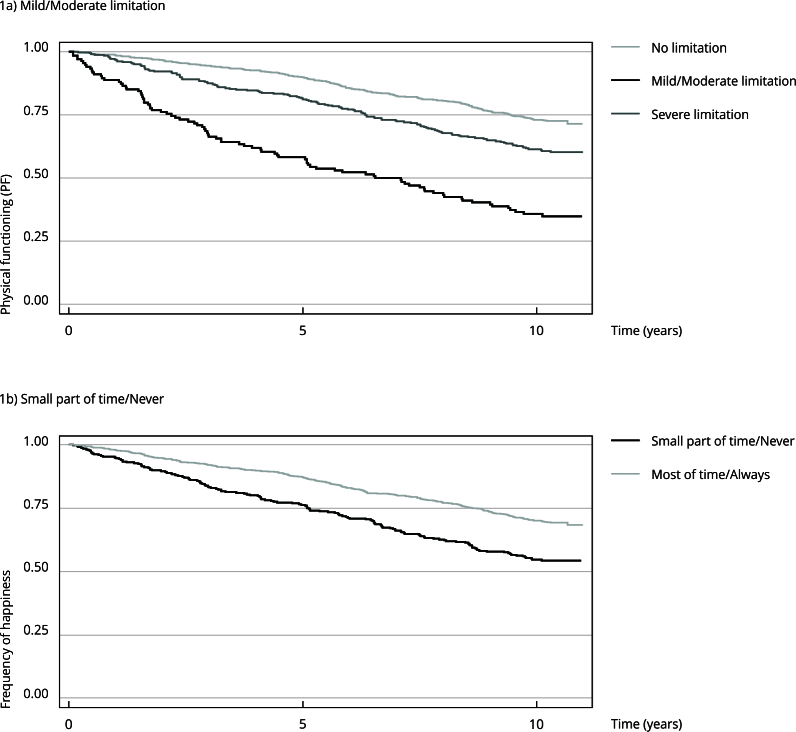
Kaplan-Meier survival curves according to functional limitations and happiness levels.

The results of the Kaplan-Meier analysis (Figures 1a and 1b) showed that survival rates were lower for individuals with severe functional limitations and those reporting lower frequency of happiness. The differences between categories were statistically significant (p < 0.05).

For physical functioning, 50% of deaths occurred within 5.2 years of follow-up among those with severe limitation, and 5.9 years among those without limitations. Regarding the feeling of happiness, 50% of deaths occurred within 5.6 years of follow-up among those who reported always or almost always feeling happy, and 4.6 years among those who reported never or almost never feeling happy (data not presented).

Table 3 (survival analysis) shows the associations between physical functioning/feeling of happiness and mortality. In the unadjusted analysis, risk of death was greater in the population with mild to moderate limitation (HR = 1.62; 95%CI: 1.29-2.03) and severe limitation (HR = 3.69; 95%CI: 2.76-4.95) compared to those with no limitations. In the model adjusted by sex, age, schooling and chronic diseases, the risk of death remains greater, with association gradient, among individuals with mild to moderate limitation (HR = 1.34; 95%CI: 1.03-1.76) and severe functional limitation (HR = 2.79; 95%CI: 2.04-3.81), compared to those with no limitations. In the unadjusted analysis, the report of happiness for a small part of the time or never increased the risk of death by 71% (HR = 1.71; 95%CI: 1.35-2.18) compared to the reference category. In the adjusted model, the risk of death was 60% greater among those who reported feeling happy a small part of the time or never (HR = 1.60; 95%CI: 1.26-2.03) compared to those who reported feeling happy more often. The results of Schoenfeld’s and global proportionality analyses demonstrated the model fit the data well.

**Table 3:** Hazard ratios (HR) and 95% confidence intervals (95%CI) of mortality in 10 years according to physical functioning and frequency of feeling of happiness. *Health Survey in Campinas Cohort* (ISA-Camp Cohort), 2008-2018.

Variables	HR	HR adjusted
(95%CI) *	(95%CI) **
Physical functioning		
No limitation	1.00	1.00
Mild to moderate limitation	1.62 (1.29-2.03)	1.34 (1.03-1.76)
Severe limitation	3.69 (2.76-4.95)	2.79 (2.04-3.81)
Frequency of feeling of happiness		
Most of the time or always	1.00	1.00
Small part of the time or never	1.71 (1.35-2.18)	1.60 (1.26-2.03)

Source: Brazilian Mortality Information System (SIM) of the Campinas Municipal Health Department.

* Unadjusted analysis;

** Adjusted by sex, age, schooling and chronic diseases.

**Figure 2:**
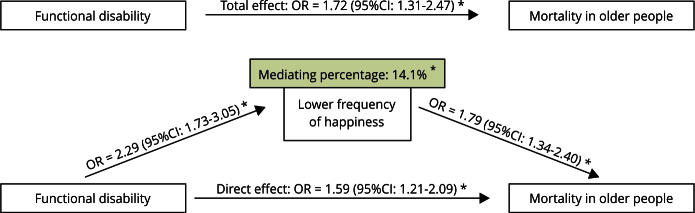
Role of frequency of happiness in the association between physical functioning and mortality in older people. *Health Survey in Campinas Cohort* (ISA-Camp Cohort), 2008-2009.

In the binary logistic regression analysis, functional disabilities increased the likelihood of a less frequent feeling of happiness by 2.29 times (OR = 2.29; 95%CI: 1.73-3.05) and mortality was more likely in the population with a less frequent feeling of happiness (OR = 1.79; 95%CI: 1.34-2.40). In the KHB analysis, the OR was 1.72 (95%CI: 1.31-2.47) for the total effect of functional disability on death and 1.59 (95%CI: 1.21-2.09) for the direct effect. The mediating percentage of the lower frequency of the feeling of happiness in the association between physical functioning and mortality was 14.1% (Figure 2).

95%CI: 95% confidence interval; OR: odds ratio. * Adjusted by sex, age, schooling and chronic diseases.

## Discussion

This study demonstrated lower survival rates among older people as limitations increase in relation to physical functioning and those who reported having feelings of happiness for a small part of the time or never. Moreover, a low frequency of happiness was a mediating factor in the association between functional disability and mortality. These findings underscore the influence of both physical functioning and subjective well-being on longevity.

Recent studies have highlighted the intricate link between functional disability ^24^ ^,^ ^26^ ^,^ ^27^ ^,^ ^29^ ^,^ ^54^, subjective well-being ^37^ ^,^ ^40^ ^,^ ^41^, and mortality in older adults, which are in line with our findings. For instance, Steptoe et al. ^40^ demonstrated that lower levels of subjective wellbeing are associated with increased mortality risk, even after controlling for physical health and other confounders. In recent findings from the *Brazilian Longitudinal Study of Aging* (ELSI-Brazil), Macinko et al. ^55^ also demonstrated significant associations of physical functioning, psychosocial and environmental factors with mortality among older adults. Specifically, having any functional limitations were associated with higher mortality risk. These findings provide crucial insights into the multifaceted nature of aging and mortality and support the need for comprehensive health strategies that address physical and psychosocial factors to promote healthy aging ^55^.

Functional disability in older adults is strongly associated with increased mortality due to several interrelated factors. Firstly, functional disability often leads to decreased physical activity, which contributes to the progression of chronic diseases such as cardiovascular diseases, diabetes, and obesity, ultimately increasing the risk of death ^56^. Secondly, individuals with functional disabilities may experience a decline in social participation, leading to isolation and increased vulnerability to mental health disorders like depression, which has been linked to higher mortality rates ^57^. Moreover, functional disability may impair the ability to perform activities of daily living, increasing the likelihood of adverse events such as falls, infections, and malnutrition, all of which significantly contribute to mortality ^58^. These interconnected factors highlight the importance of maintaining functional capacity to increase survival among older adults.

The association between happiness and mortality has been documented in international studies ^40^ ^,^ ^41^. In a study involving a representative sample of the British population, researchers found a significant reduction in the risk of premature death throughout the follow-up period among older people who reported better subjective well-being ^40^. This protective effect of happiness remained significant even after controlling for age, sex, socioeconomic status and preexisting health conditions. A systematic review and meta-analysis investigated the effect of positive feelings, including happiness, on health and mortality in longitudinal studies ^41^. The results showed that greater positive affectivity was associated with a significant reduction in the risk of premature mortality in older people regardless of age, sex, physical health, and behavioral risk factors. 

With additional years of life, older people have a greater chance of experiencing important moments, such as celebrating birthdays, participating in family events, achieving personal goals and sharing knowledge with younger generations, thus preserving culture and history ^37^. Happier older people tend to have better physical and mental health, as happiness is associated with decreased chronic health problems, as well as greater resilience, hope and harmony ^59^. Optimism and positivity can positively influence the immune system and reduce stress and anxiety, being associated with a healthier lifestyle (such as healthier eating habits, practicing regular physical exercises) and avoiding harmful behaviors (such as smoking and excessive alcohol intake) ^5^ ^,^ ^60^. Happiness is also associated with a solid social support network. Older people who feel loved, supported and connected to their families, friends and community have a greater sense of purpose and meaning of life, which can contribute to greater longevity ^61^. Mental health conditions can also influence health-related decision-making, such as adhering to medical treatment and searching for preventive healthcare. Lack of motivation resulting from a greater frequency of unhappy moments can trigger behaviors and reactions that increase the risk of health complications and, consequently, premature death ^62^.

A strong association was found between physical functioning and the feeling of happiness, which is in line with previous studies ^5^ ^,^ ^35^. A possible explanation for these findings is the reduction in independence, autonomy and freedom in the population living with functional limitations, which can trigger feelings of anguish, unsatisfaction and unhappiness. Moreover, a lower frequency of happiness was a mediator in the negative impact of functional disability on mortality, as a lower frequency of happy moments increased the risk of premature death by 14% in the older population living with disabilities. These findings underscore the need to care for older people with limitations, not only regarding physical aspects, but also given the impact of such conditions on mental health. These relationships also illustrate how emotional dimensions can have profound repercussions in various spheres of health. By considering a lower frequency of happiness a mediating factor in the negative impact of functional disability on longevity, one recognizes that emotions can shape physiological and behavioral aspects in a tangible way ^63^.

### Limitations and strengths

Caution is needed when interpreting the association between the feeling of happiness and functional disability, as the study design does not allow for causal inferences. Functional disability develops over the life course of older adults, whereas happiness is a momentary feeling, suggesting that disabilities are more likely to lead to unhappiness, rather than the inverse. We also recognize the limitation of not using specific validated scales to measure functional disability; instead, we relied on items from the SF-36 questionnaire. Similarly, we did not use a specific scale to measure happiness, opting for a single question about frequency of the feeling of happiness over the past four weeks, with five possible response categories, which is suitable for large-scale studies ^64^. Evaluating the frequency of happiness rather than its intensity provides a significant advantage, as frequency is more likely to capture the stability of subjective well-being over time ^5^ ^,^ ^64^ ^,^ ^65^.

Notably, this study also has strengths. We used a representative sample of the population of Campinas studied over a 10-year period with fundamental variables for the control of confounding. Moreover, this study offers unprecedented results in Brazil and Latin America. No cohort studies were found in the Brazilian literature that directly address the relationship between happiness and mortality in older people. The continuity of research in this field could lead to a deeper understanding of mechanisms underlying this association and enable the generalization of these results to different cultural and socioeconomic contexts.

### Health care, clinical and policy implications

This study underscores the importance of good physical functioning and happiness in the longevity of individuals aged 60 years or older, revealing the role of happiness in the association between physical functioning and mortality.

Providing care for older people with a multidisciplinary team, including physical therapy and psychotherapy sessions, is essential, with special attention to low-income populations and those served by the Brazilian Unified National Health System (SUS) to ensure the incorporation of these strategies into their care. It is also crucial to emphasize the importance of remaining physically and mentally active throughout life to promote better health outcomes in old age. The incorporation of indicators of physical limitations and well-being, including happiness, into the health diagnosis of older people could contribute to creating clinical and policy strategies. Thus, it will be possible not only to improve the quality of life of older people, but also to promote greater longevity, reinforcing the importance of a comprehensive health approach that considers the various dimensions of health, especially physical and emotional aspects.
